# Supplementary material for: Predictors of severe or lethal COVID-19, including Angiotensin Converting Enzyme inhibitors and Angiotensin II Receptor Blockers, in a sample of infected Italian citizens
Source: PLoS One. 2020 Jun 24;15(6):e0235248. doi: 10.1371/journal.pone.0235248 (PMC7314008; doi:10.1371/journal.pone.0235248)
Supplement: Attachment — Submitted filename: Response to Reviewers.doc [file pone.0235248.s002.doc]

**Answers to Referee I comments**

I-1. The Referee wrote: “Please clarify the novelty of this study. Especially, how about discussing a little bit more the strength and shortness of this study compared with a report by Mehra et al.?"

We agree that, unfortunately, the results are not novel. Please acknowledge, however, that the evidence on SARS-CoV-2 is emerging so rapidly that a finding becomes old after weeks. Please believe that we are the first to be sorry for that. We also entirely agree that the strengths and weaknesses of the study should have been discussed further, comparing it with Mehra et al. and the other observational studies on the same topic (which now also include Mehta et al., JAMA Cardiol 2020). Accordingly, to comply also with the suggestions of the first reviewer, we entirely revised the Discussion section. In specific, the previous paragraphs:

"Recently, three large observational studies based upon inpatient only [14] or in- and outpatient electronic health records [15, 16] examined the association between antihypertensive medications and the risk of COVID-19 and/or a severe/lethal disease: our results substantially confirm these previous findings and, concordantly, no increased risk emerged with ACE inhibitors and/or ARBs use. Of note, Mehra et al [14], after controlling for age, gender, current smoke and comorbidities, found similar death rates among COVID-19 patients using ARBs, and even lower death rates among ACE inhibitors users. The latter results, however - although in line with a previous observational analysis on Chinese in-hospital COVID-19 subjects [12], are based upon hospitalized patients only, may be due to unmeasured confounding and, in the absence of randomized evidence, should be considered only preliminary [23].

Our study, likewise Mancia et al and Reynolds at al [16], included all infected subjects (either hospitalized or not), retrieved all medications and admissions from electronic health records, and evaluated the disease progression beyond mortality. Concordantly, all these findings confirm those from preliminary Chinese cohorts [12, 19] and strongly support the statements of several experts [24, 25] and scientific societies, including the European Medicines Agency [26], the European Society of Cardiology [27], and the American Heart Association [28], who recommend continuation of ARBs or ACE inhibitors medication for all patients, unless otherwise advised by their physicians."

have been replaced by the followings:

"Recently, a few large observational studies based upon inpatient only [26] or in- and outpatient electronic health records [27-29] examined the association between antihypertensive medications and the risk of COVID-19 and/or a severe/lethal disease: our results are in line with most of the previous findings on an absence of risk with ACE inhibitors and/or ARBs use. In brief, the studies by Mancia et al [27], and Reynolds et al [28] reported no difference in COVID-19 severity or death between treated and untreated subjects, for both ARBs and Ace inhibitors. Instead, the results by Mehra et al [26] and Mehta et al [29] were partially discordant: both studies found no association between treatment with ARBs and death, but those treated with ACE inhibitors showed a significantly higher risk of ICU admission in the study by Mehta et al [29], and a significantly lower risk of in-hospital death in the study by Mehra et al. [26]. In our sample, in order to further investigate the potential beneficial effects of ACE inhibitors, the impact of which might be larger in patients with diabetes, COPD, or cardiovascular diseases, we performed additional analyses, stratified by comorbidities. We found however no significant differences in the risk of severe/lethal COVID-19 among treated and untreated patients with either CVD, or diabetes and COPD (data not shown).

Besides sample size and provenance, the main differences between this and previous studies pertain the inclusion criteria (we included all subjects, hospitalized or not, such as Mancia et al [27], Reynolds et al [28], Mehta et al [29], and unlike Mehra et al [26]); the retrieval of all medications and admissions from electronic health records (unlike Mehta et al [29]), and the evaluation of disease progression beyond mortality (unlike Mehra et al [26]).

Overall, the present and previous findings confirm those from preliminary Chinese cohorts [24, 32], and although confirmation from randomized studies is required [39], they strongly support the statements of several experts [40, 41] and scientific societies, including the European Medicines Agency [42], the European Society of Cardiology [43], and the American Heart Association [44], who recommend continuation of ARBs or ACE inhibitors medication for all patients, unless otherwise advised by their physicians."

I-2. The Referee wrote: “If the data are available, how about comparing the prevalence of hypertension, CVD, and diabetes between the diseased subjects and the whole residence of Provinces of Ferrara and Pescara or Italy?"

We agree and accordingly added the following sentences in the Results section: "The sample of infected subjects, compared with the available estimates of the general population of the two provinces [34], showed higher prevalence of diabetes (12.8% in the sample versus ≅5% in the general population); hypertension (33.9% versus ≅17%), COPD (6.0% versus ≅3%) and CVD (16.1% versus ≅3%). Please acknowledge that, although the estimates of the two provinces were quite similar (with the exception of cardiovascular diseases), the overall estimate from the provinces was derived computing a weighted mean of the latest available data (2018) from the Italian Institute of Statistics.

I-3. The Referee wrote: “Table 1. Please use 2-way ANOVA followed by post-hoc analysis for the comparison among 3 groups stratified by the COVID-19 severity".

We agree and we accordingly used two-way ANOVA followed by Tukey honestly significant difference test. Methods and Table footnotes were updated, while the Results did not differ.

I-4. The Referee wrote: “Please clarify each variable included in multivariate logistic regression analysis, instead of the current description: “All recorded variables were included a priori in all models, …”

We agree and we accordingly added that age, gender, CVD, diabetes, renal disease, cancer and COPD were included a priori in all models.

I-5. The Referee wrote: “Figure 1 may not be necessary".

We agree and accordingly cut Figure 1.

I-6. The Referee wrote: “Lines 170-171. The gist of the description is unclear and misleading."

We agree and we accordingly revised the text. The following sentences:

"On one side, some asked whether the therapy should be discontinued during SARS-Cov-2 pandemic [7, 22] because of a strong association between hypertension and disease severity (although the pharmacological treatment were not assessed) [2-4], and based on the hypothesis that (a) ACE2 up-regulation mediated by ARBs (and, to a lesser extent, by ACE inhibitors) might increase patients' susceptibility to SARS-CoV-2 entry into host cells and further viral propagation [20], (b) virus binding to ACE2 might reduce its activity, thus leading to increased levels of Angiotensin II and consequent pulmonary vasoconstriction, inflammation and oxidative organ damage, and increased risk of acute lung injury [8]"

were replaced with

"On one side, some asked whether the therapy should be discontinued during SARS-Cov-2 pandemic [17, 38] because COVID-19 was strongly associated with hypertension, which is frequently treated with ARBs or ACE inhibitors [2-4]. It was indeed hypothesized that (a) ACE2 up-regulation mediated by ARBs (and, to a lesser extent, by ACE inhibitors) might increase patients' susceptibility to SARS-CoV-2 entry into host cells and further viral propagation [18, 19], (b) virus binding to ACE2 might reduce its activity, thus leading to increased levels of Angiotensin II and consequent pulmonary vasoconstriction, inflammation and oxidative organ damage, and increased risk of acute lung injury [20]."

I-7. The Referee wrote: “Ref 5,6 were not adequate."

We agree, and we accordingly replaced references n. 5-6 with the following (now numbered as [5-8]):

5. Hoffmann M, Kleine-Weber H, Schroeder S, et al. SARSCoV-2 cell entry depends on ACE2 and TMPRSS2 and is blocked by a clinically proven protease inhibitor. Cell 2020;181:271-80.

6. Kuba K, Imai Y, Rao S, Gao H, Guo F, Guan B, et al. A crucial role of angiotensin converting enzyme 2 (ACE2) in SARS coronavirus-induced lung injury. Nat Med. 2005;11(8):875-9.

7. Li W, Moore MJ, Vasilieva N, et al. Angiotensin-converting enzyme 2 is a functional receptor for the SARS coronavirus. Nature 2003;426: 450-4.

8. Xiao X, Chakraborti S, Dimitrov AS, Gramatikoff K, Dimitrov DS. The SARS-CoV S glycoprotein: expression and functional characterization. Biochem Biophys Res Commun. 2003;312(4):1159-64

Please also acknowledge that the sentences in the Introduction previously reported as:

"Observing that human pathogenic coronaviruses bind their target cells through angiotensin-converting enzyme 2 (ACE2) [5], and that the expression of ACE2 is substantially increased in diabetics and patients who are treated with ACE inhibitors and angiotensin II type-I receptor blockers (ARBs) [5,6], Fang and colleagues hypothesized that the increased expression of ACE2 would facilitate infection with Severe Acute Respiratory Syndrome Coronavirus 2 (SARS-CoV-2), thus the hypertension treatment with ACE2-stimulating drugs, as well as ACE2 polymorphisms, might increase the risk of developing severe COVID-19 [7]"

were replaced as follows:

"Observing that human pathogenic coronaviruses bind their target cells through angiotensin-converting enzyme 2 (ACE2) [5-8], and that a few studies reported an increase in ACE2 expression mediated by angiotensin II type-I receptor blockers (ARBs) and ACE inhibitors (more consistently on animals than in humans) [9-16], some hypothesized that the increased expression of ACE2 would facilitate infection with Severe Acute Respiratory Syndrome Coronavirus 2 (SARS-CoV-2), thus the hypertension treatment with ACE2-stimulating drugs, as well as ACE2 polymorphisms, might increase the risk of developing severe COVID-19 [17-19]."

We accordingly added the following new references:

9. Furuhashi M, Moniwa N, Mita T, Fuseya T, Ishimura S, Ohno K, et al. Urinary angiotensin-converting enzyme 2 in hypertensive patients may be increased by olmesartan, an angiotensin II receptor blocker. Am J Hypertens. 2015;28(1):15-21.

10. Luque M, Martin P, Martell N, Fernandez C, Brosnihan KB, Ferrario CM. Effects of captopril related to increased levels of prostacyclin and angiotensin-(1-7) in essential hypertension. J Hypertens. 1996;14(6):799-805.

11. Zisman LS, Keller RS, Weaver B, Lin Q, Speth R, Bristow MR, et al. Increased angiotensin-(1-7)-forming activity in failing human heart ventricles: evidence for upregulation of the angiotensin-converting enzyme Homologue ACE2. Circulation. 2003;108(14):1707-12.

12. Ferrario CM, Jessup J, Chappell MC, Averill DB, Brosnihan KB, Tallant EA, et al. Effect of angiotensin-converting enzyme inhibition and angiotensin II receptor blockers on cardiac angiotensin-converting enzyme 2. Circulation. 2005;111(20):2605-10.

13. Gallagher PE, Ferrario CM, Tallant EA. MAP kinase/phosphatase pathway mediates the regulation of ACE2 by angiotensin peptides. Am J Physiol Cell Physiol. 2008;295(5):C1169-74.

14. Igase M, Strawn WB, Gallagher PE, Geary RL, Ferrario CM. Angiotensin II AT1 receptors regulate ACE2 and angiotensin-(1-7) expression in the aorta of spontaneously hypertensive rats. Am J Physiol Heart Circ Physiol. 2005;289(3):H1013-9.

15. Ishiyama Y, Gallagher PE, Averill DB, Tallant EA, Brosnihan KB, Ferrario CM. Upregulation of angiotensin-converting enzyme 2 after myocardial infarction by blockade of angiotensin II receptors. Hypertension. 2004;43(5):970-6.

16. Soler MJ, Ye M, Wysocki J, William J, Lloveras J, Batlle D. Localization of ACE2 in the renal vasculature: amplification by angiotensin II type 1 receptor blockade using telmisartan. Am J Physiol Renal Physiol. 2009;296(2):F398-405.

18. Esler M, Esler D. Can angiotensin receptor-blocking drugs perhaps be harmful in the COVID-19 pandemic? J Hypertens 2020;38:781-2.

19. Sommerstein R, Grani C. Preventing a COVID-19 pandemic: ACE inhibitors as a potential risk factor for fatal COVID-19. BMJ 2020;368: m810.

**Answers to Referee II comments**

II-1. The Referee wrote: “The authors conducted retrospective case-control study in Italian citizens trying to identify potential independent predictors of severe/lethal COVID-19, and showed no association between COVID-19 severity and treatment with ARBs and/or ACE inhibitors, supporting the previous recommendation to continue the medication. This is an important topic, but it was unfortunate not to see any new findings. The diagnosis of COVID-19 should be confirmed by PCR tests. Although they described where the laboratory diagnosis was done, they did not mention PCR tests at all. They should provide detailed information of PCR tests."

We entirely agree that his information should have been reported, and we are sorry for the oversight. We accordingly added the following sentences in the Methods section: "All diagnoses were made using (real time) reverse transcription polymerase chain reaction (rRT-PCR) on oropharingeal specimens. The assays were those originally proposed by the Charité-Universitätsmedizin Berlin Institute of Virology [33], and then endorsed by the WHO [34]".

The following references were thus added:

33. Corman VM, Landt O, Kaiser M, Molenkamp R, Meijer A, et al. (2020) Detection of 2019 novel coronavirus (2019-nCoV) by real-time RT-PCR. Euro Surveill 25.

34. World Health Organization (2020) Laboratory testing for coronavirus disease (COVID-19) in suspected human cases - Interim guidance.

II-2. The Referee wrote: “The worse symptoms of cardiovascular diseases would increase the opportunity to use ARBs or ACE inhibitors. Normalization of the data with disease severity would be necessary. In addition, treatment with ACE inhibitors or ARBs should be compared with other anti-hypertensive drugs."

Please acknowledge that this point is conceptually related to points II-3 and II-4, and they have thus been discussed together. We entirely agree that the worse symptoms of cardiovascular diseases may increase the opportunity to use ARBs or ACE inhibitors, thus RAAS inhibitors on diabetes patients might have protective impacts on COVID-19 severity. And we thus agree that beneficial or adverse effects of the drugs should have been examined more rigorously.

Accordingly, in order to investigate whether RAAS inhibitors might have had protective impacts among the patients with diabetes, COPD and CVD, we stratified the analyses on the potential association between RAAS inhibitors and COVID-19 severity by CVD, COPD, and diabetes. Please find below the results on the sample of hypertensive subjects.

| Antihypertensive treatment with: | Mild | Severe | Very severe/lethal |
| --- | --- | --- | --- |
| Variables |  |  |  |
|  |  |  |  |
| Stratified by CVD |  |  |  |
| Without CVD | (n=155) | (n=106) | (n=69) |
| - ACE inhibitors, % | 54.2 | 45.1 | 49.3 |
| - ARBs, % | 41.9 | 46.2 | 42.0 |
| - ACE inhibitors or ARBs, % | 91.0 | 80.2 | 88.4 |
|  |  |  |  |
| With CVD | (n=52) | (n=101) | (n=60) |
| - ACE inhibitors, % | 44.2 | 43.6 | 36.7 |
| - ARBs, % | 40.4 | 40.6 | 38.3 |
| - ACE inhibitors or ARBs, % | 80.8 | 77.2 | 71.7 |
|  |  |  |  |
|  |  |  |  |
| Stratified by diabetes |  |  |  |
| Without diabetes | (n=165) | (n=151) | (n=88) |
| - ACE inhibitors, % | 55.2 | 45.7 | 44.3 |
| - ARBs, % | 38.8 | 39.7 | 34.1 |
| - ACE inhibitors or ARBs, % | 89.1 | 78.8 | 76.2 |
|  |  |  |  |
| With diabetes | (n=42) | (n=56) | (n=41) |
| - ACE inhibitors, % | 38.1 | 33.9 | 41.5 |
| - ARBs, % | 52.4 | 53.6 | 53.7 |
| - ACE inhibitors or ARBs, % | 85.7 | 78.6 | 90.2 |
|  |  |  |  |
|  |  |  |  |
| Stratified by COPD |  |  |  |
| Without COPD | (n=194) | (n=179) | (n=110) |
| - ACE inhibitors, % | 53.1 | 44.7 | 44.6 |
| - ARBs, % | 40.7 | 44.7 | 42.7 |
| - ACE inhibitors or ARBs, % | 88.7 | 81.6 | 83.6 |
|  |  |  |  |
| With COPD | (n=13) | (n=28) | (n=19) |
| - ACE inhibitors, % | 30.8 | 28.6 | 36.8 |
| - ARBs, % | 53.9 | 35.7 | 26.3 |
| - ACE inhibitors or ARBs, % | 84.6 | 60.7 | 63.2 |
|  |  |  |  |

Among patients with diabetes, COPD or CVD, the use of RAAS inhibitors was not significantly associated with COVID-19 severity. We accordingly added the following sentences in the Discussion section: " In our sample, in order to further investigate the potential beneficial effects of ARBs and ACE inhibitors, the impact of which might be larger in patients with diabetes, COPD, or cardiovascular diseases, we performed additional analyses, stratified by comorbidities. We found however no significant differences in the risk of severe/lethal COVID-19 among treated and untreated patients with either CVD, or diabetes and COPD (data not shown)".

We also agree that it may be of interest to compare ACE inhibitors or ARBs with other anti-hypertensive drugs. However, unfortunately, we could not have the data, as the original protocol focused only on ARBs or ACE inhibitors, and the data on other anti-hypertensive drugs were therefore not collected.

II-3. The Referee wrote: “The authors compared with or without ARBs / ACE inhibitors in hypertension. Sometimes diseases are overlapped. They should clarify whether diabetes or COPD are overlapped with cardiovascular diseases. RAS inhibitors on diabetes patients might have protective impacts on COVID-19 severity."

We agree that some overlapping between cardiovascular diseases and diabetes or COPD exists. Also, we agree that the potential protective effect of RAAS inhibitors among the patients with diabetes or other comorbidities should have been investigated further (as suggested in the following point). We accordingly performed additional, stratified analyses in order to investigate whether RAAS inhibitors might have had protective impacts among the patients with diabetes, COPD and CVD, separately. Please acknowledge that this is issue is conceptually related to issues II-2 and II-3 and was discussed after point II-2.

Concerning the potential overlapping between CVD and COPD or diabetes, this was certainly the case, as shown below. However, please acknowledge that the overlapping was not as high as to cause multicollinearity. It was therefore possible to perform multivariable analyses, adjusting for the potential influence of CVD on the association between RAAS inhibitors and COVID-19 among the subjects with diabetes and COPD.

```
           |         Diabetes
       CVD |         0          1 |     Total
-----------+----------------------+----------
         0 |     1,224        121 |     1,345
         1 |       185         73 |       258
-----------+----------------------+----------
     Total |     1,409        194 |     1,603


           |         COPD
       CVD |         0          1 |     Total
-----------+----------------------+----------
         0 |     1,297         48 |     1,345
         1 |       209         49 |       258
-----------+----------------------+----------
     Total |     1,506         97 |     1,603
```

II-4. The Referee wrote: “It is interesting to see the ACE Inhibitors or ARBs treatment showing a trend for reduced odds ratio (Table 2). Beneficial or adverse effects of the drugs would have to be examined more rigorously. For instance, was prescription or medication continued during the infection for each patient?"

We entirely agree that we should have clarified this issue, and try to investigate further the potential beneficial effects of the drugs on COVID-19. With regard to medication, we added in the text that the treatment with ARBs or ACE inhibitors was not discontinued for any participant. Concerning the potential beneficial effect of the drugs, we performed additional, stratified analyses in order to investigate whether RAAS inhibitors might have had protective impacts among the patients with diabetes, COPD and CVD, separately. Please acknowledge that this is issue is conceptually related to issues II-2 and II-3 and was discussed after point II-2.

II-5. The Referee wrote: “For references, they cited recent short review or commentary on COVID-19 but mostly ignored key original papers. This is not acceptable. There are nearly two decades of research on ACE2 and SARS-CoV accumulated, and both basic and clinical papers of origin should be cited. The reference should be extensively revised."

We agree and thank the Referee for the suggestion. We accordingly replaced the references previously numbered as 5-6 with the followings (now numbered as [5-8]):

5. Hoffmann M, Kleine-Weber H, Schroeder S, et al. SARSCoV-2 cell entry depends on ACE2 and TMPRSS2 and is blocked by a clinically proven protease inhibitor. Cell 2020;181:271-80.

6. Kuba K, Imai Y, Rao S, Gao H, Guo F, Guan B, et al. A crucial role of angiotensin converting enzyme 2 (ACE2) in SARS coronavirus-induced lung injury. Nat Med. 2005;11(8):875-9.

7. Li W, Moore MJ, Vasilieva N, et al. Angiotensin-converting enzyme 2 is a functional receptor for the SARS coronavirus. Nature 2003;426: 450-4.

8. Xiao X, Chakraborti S, Dimitrov AS, Gramatikoff K, Dimitrov DS. The SARS-CoV S glycoprotein: expression and functional characterization. Biochem Biophys Res Commun. 2003;312(4):1159-64

Finally, please acknowledge that we also added the following references:

9. Furuhashi M, Moniwa N, Mita T, Fuseya T, Ishimura S, Ohno K, et al. Urinary angiotensin-converting enzyme 2 in hypertensive patients may be increased by olmesartan, an angiotensin II receptor blocker. Am J Hypertens. 2015;28(1):15-21.

10. Luque M, Martin P, Martell N, Fernandez C, Brosnihan KB, Ferrario CM. Effects of captopril related to increased levels of prostacyclin and angiotensin-(1-7) in essential hypertension. J Hypertens. 1996;14(6):799-805.

11. Zisman LS, Keller RS, Weaver B, Lin Q, Speth R, Bristow MR, et al. Increased angiotensin-(1-7)-forming activity in failing human heart ventricles: evidence for upregulation of the angiotensin-converting enzyme Homologue ACE2. Circulation. 2003;108(14):1707-12.

12. Ferrario CM, Jessup J, Chappell MC, Averill DB, Brosnihan KB, Tallant EA, et al. Effect of angiotensin-converting enzyme inhibition and angiotensin II receptor blockers on cardiac angiotensin-converting enzyme 2. Circulation. 2005;111(20):2605-10.

13. Gallagher PE, Ferrario CM, Tallant EA. MAP kinase/phosphatase pathway mediates the regulation of ACE2 by angiotensin peptides. Am J Physiol Cell Physiol. 2008;295(5):C1169-74.

14. Igase M, Strawn WB, Gallagher PE, Geary RL, Ferrario CM. Angiotensin II AT1 receptors regulate ACE2 and angiotensin-(1-7) expression in the aorta of spontaneously hypertensive rats. Am J Physiol Heart Circ Physiol. 2005;289(3):H1013-9.

15. Ishiyama Y, Gallagher PE, Averill DB, Tallant EA, Brosnihan KB, Ferrario CM. Upregulation of angiotensin-converting enzyme 2 after myocardial infarction by blockade of angiotensin II receptors. Hypertension. 2004;43(5):970-6.

16. Soler MJ, Ye M, Wysocki J, William J, Lloveras J, Batlle D. Localization of ACE2 in the renal vasculature: amplification by angiotensin II type 1 receptor blockade using telmisartan. Am J Physiol Renal Physiol. 2009;296(2):F398-405.

18. Esler M, Esler D. Can angiotensin receptor-blocking drugs perhaps be harmful in the COVID-19 pandemic? J Hypertens 2020;38:781-2.

19. Sommerstein R, Grani C. Preventing a COVID-19 pandemic: ACE inhibitors as a potential risk factor for fatal COVID-19. BMJ 2020;368: m810.
