# Supplementary material for: Predictors of severe or lethal COVID-19, including Angiotensin Converting Enzyme inhibitors and Angiotensin II Receptor Blockers, in a sample of infected Italian citizens
Source: PLoS One. 2020 Jun 24;15(6):e0235248. doi: 10.1371/journal.pone.0235248 (PMC7314008; doi:10.1371/journal.pone.0235248)
Supplement: Attachment — Submitted filename: Response to Reviewers.doc [file pone.0235248.s003.doc]

**Answers to Editor's comments**

E-1

The Editor wrote: "Please provide statistical power analysis as reviewer 2 requested".

We agree and we accordingly added the following paragraph in the Methods section: "Our sample of 129 hypertensive subjects with severe/lethal COVID-19, and 414 hypertensive subjects with mild disease or asymptomatic infection, had 80% statistical power to detect a difference of 20% or higher (corresponding to a relative risk ≥1.20) in the risk of severe/lethal death between the users of ARBS or ACE inhibitors (exposed group), and non users (controls)".

**Answers to Referee I comments**

I-1. The Referee wrote: "The authors have adequately responded to all queries I raised. The manuscript has been improved very much".

We thank the Referee for his comment.

**Answers to Referee II comments**

II-1. The Referee wrote: "Comparison with other anti-hypertensive drugs is essential to precisely determine the effects of ACE inhibitors or ARBs on COVID-19 severity and lethality. In addition to COVID-19, the severity of underlying cardiovascular diseases should be also included. As such, it revealed that number and information of the patients in this study was not enough to reach their conclusion".

On one side, we agree that, hypothetically, both the severity of cardiovascular diseases and the use of other antihypertensive medications could be important to discern more precisely the relationship between ARBS or ACE inhibitors and COVID-19. On the other side, however, please acknowledge that:

1.

None of the four studies published on the topic measured the severity of underlying cardiovascular diseases (Mehta, JAMA Cardiol 2020; Mancia, N Engl J Med 2020; Reynolds, N Engl J Med 2020; Mehra, N Engl J Med 2020). Thus, this remains a plausible but unverified hypothesis and, as happened for the above studies, it cannot cancel the validity of the present study.

2.

Concerning the use of other antihypertensive drugs, of the four studies published on the topic, one did not report these data (Mehta, JAMA Cardiol 2020), one study reported the information but did not include this in multivariable analyses (Mehra, N Engl J Med 2020), and the other two (Mancia, N Engl J Med 2020; Reynolds, N Engl J Med 2020) collected these data and included them in multivariable analysis. In both cases, however, there were no substantial differences between the adjusted and unadjusted relative risks of death of ARBS or ACE inhibitors (which suggests that the additional covariates of the models, including other antihypertensive medications, did not impact alter noticeably the relationship between severity and use of ARBS or ACE inhibitors). This is also supported by the fact that our results were in line with those of the two studies from Mancia et al., and Reynolds et al. Therefore, also this hypothesis, although plausible, remains unverified.

Since it is not proven that both the severity of underlying cardiovascular diseases and use of other antihypertensive drugs may confound, or even mediate, the relationship between COVID-19 and use of ARBS or ACE inhibitors, the validity of the study cannot be nullified by the absence of an evaluation of these hypotheses. We do agree, however, that both hypotheses are potential limitations, and they should have been acknowledged in the text. Accordingly, the following sentences were added in the limitations section:

"Other limitations are the lack of an evaluation of the severity of the underlying cardiovascular diseases, and the absence of data on other antihypertensive medications. However, their potential role in altering the relationship between ARBS or ACE inhibitors and risk of severe/lethal COVID-19 remains unclear: none of the previous studies on the topic assessed cardiovascular diseases severity [26-29], and the two studies that included the use of other antihypertensive drugs into multivariable analyses did not find substantial differences between the adjusted and unadjusted relative risks of death [27,28]".

II-2. The Referee wrote: "The authors did not made all data underlying the findings in their manuscript fully available".

Please acknowledge that this is incorrect: we uploaded the entire dataset - named Appendix S1 - as online supporting information since the initial submission, to comply with PLOS One data availability requirement. Please acknowledge that, at the end of the manuscript, under the "Access to data" section, we reported the following sentence: "The complete dataset used in this work is available in Appendix S1".
