# Supplementary material for: Predictors of severe or lethal COVID-19, including Angiotensin Converting Enzyme inhibitors and Angiotensin II Receptor Blockers, in a sample of infected Italian citizens
Source: PLoS One. 2020 Jun 24;15(6):e0235248. doi: 10.1371/journal.pone.0235248 (PMC7314008; doi:10.1371/journal.pone.0235248)
Supplement: Attachment — Submitted filename: Response to Reviewers.doc [file pone.0235248.s004.doc]

**Answers to Editor's comments**

E-1. The Editor wrote: "The authors responded adequately, however, the reference 26 is retracted.  Please edit the manuscript without citing this article".

We agree and we thank the Editor for the comment. Accordingly, we deleted all the citations to the retracted article throughout the text. Please acknowledge that, in place of the above reference, we added the following new reference for some of the sentences in the Discussion:

44. Williamson E, Walker AJ, Bhaskaran K, Bacon S, Bates C, Morton CE, et al. OpenSAFELY: factors associated with COVID-19-related hospital death in the linked electronic health records of 17 million adult NHS patients. MedRxiv. 2020. doi: https://doi.org/10.1101/2020.05.06.20092999.

E-2. The Editor also wrote: "Also the information of reference 27 is not completed".

We agree and accordingly updated and completed the reference. Please accept our apologies for the oversight.
